# Physiology of microalgae and their application to sustainable agriculture: A mini-review

**DOI:** 10.3389/fpls.2022.1005991

**Published:** 2022-11-17

**Authors:** Iffet Çakirsoy, Takuji Miyamoto, Norikuni Ohtake

**Affiliations:** ^1^ Graduate School of Science and Technology, Niigata University, Niigata, Japan; ^2^ Sakeology Center, Niigata University, Niigata, Japan

**Keywords:** microalga, sustainable agriculture, nutrient recycling, fertilizer alternative, CO_2_-concentrating mechanism, membrane lipid remodeling

## Abstract

Concern that depletion of fertilizer feedstocks, which are a finite mineral resource, threatens agricultural sustainability has driven the exploration of sustainable methods of soil fertilization. Given that microalgae, which are unicellular photosynthetic organisms, can take up nutrients efficiently from water systems, their application in a biological wastewater purification system followed by the use of their biomass as a fertilizer alternative has attracted attention. Such applications of microalgae would contribute to the accelerated recycling of nutrients from wastewater to farmland. Many previous reports have provided information on the physiological characteristics of microalgae that support their utility. In this review, we focus on recent achievements of studies on microalgal physiology and relevant applications and outline the prospects for the contribution of microalgae to the establishment of sustainable agricultural practices.

## Introduction

With the increasing threat of mineral resource depletion through human activities, demand for renewable feedstocks is rising dramatically. The utilization of photosynthetic organisms, including land plants and algae, offers one promising solution. For example, lignocellulosic biomass, which is composed predominantly of plant secondary cell walls, represents an abundant and renewable feedstock for materials, chemicals, and fuels (Ragauskas et al., 2014; Umezawa, 2018; Miyamoto et al., 2020). Promoting the applications of photosynthetic organisms would contribute to the establishment of a sustainable human society.

In the context of agricultural sustainability, a renewable alternative to synthetic chemical fertilizers is urgently required. Enhanced utilization of synthetic chemical fertilizers in conjunction with the development of modern crop cultivars, in which the yield is highly responsive to intensive fertilization, has contributed to improved crop productivity worldwide (Khush, 2001). For example, in soils a large portion of phosphorus (P), an essential macronutrient for plants, likely exists as non-available or poorly available forms for crops, which increases the importance of P fertilizer. However, because the raw material of P fertilizers, rock phosphate, is a finite resource distributed unevenly in limited areas of the world, depletion of the reserves is of grave concern (Desmidt et al., 2015). In addition, the manufacture of nitrogen (N) fertilizers requires the burning of fossil fuels to fix atmospheric N_2_ and intensive use of N fertilizers enriches reactive N compounds, leading to soil acidification, water eutrophication, and atmospheric pollution (Hayashi et al., 2021). Thus, to establish a sustainable agricultural system worldwide, renewable alternatives to chemical fertilizers and the adoption of eco-friendly soil fertilization practices (Lin et al., 2019), as well as strategies to increase the nutrient use efficiency of crops (Hu et al., 2015; Wu et al., 2020; Ochiai et al., 2022), should be explored.

Microalgae are unicellular photosynthetic organisms commonly found in freshwater and marine ecosystems. They have been used in both experimental and real-world settings to biologically purify wastewater (Vadiveloo et al., 2021). Wastewater purification systems using microalgae represent a promising alternative to conventional wastewater treatment technologies that consume high amounts of energy, discharge sludge, and emit greenhouse gases (Qiao et al., 2020). Microalgae can rapidly grow and proliferate by efficiently acquiring carbon dioxide (CO_2_) and nutrients, such as P and N, from water systems (Sukačová et al., 2020). Also, the use of microalgal biomass as a biofertilizer as well as a fuel resource can contribute to the enhanced recycling of nutrients (Das et al., 2019; Khan et al., 2019; Moges et al., 2020).

Previous works have revealed many physiological characteristics favorable to the use of microalgae in sustainable agriculture. In addition, empirical evidence on the effectiveness and characteristics of microalga-based fertilizers associated with their physiology has been reported. This review is focused on interactions between basic and applied studies of microalgae, providing insight into a strategy for the establishment of sustainable agriculture.

## Carbon fixation capacity assisted by CO_2_-concentrating mechanisms

The CO_2_ assimilation capacity of photosynthetic organisms is critical to their growth. Ribulose 1,5-bisphosphate carboxylase/oxygenase (Rubisco) is a core enzyme involved in carbon fixation reactions. However, Rubisco generally shows a low affinity for CO_2_ and the carboxylation reaction has a slow catalytic turnover rate. The oxygenase activity of Rubisco is also associated with CO_2_-consuming photorespiration. These properties of Rubisco limit the efficiency of carbon fixation in photosynthetic organisms. In addition to the properties of Rubisco, aquatic conditions present further challenges for algal carbon fixation because the diffusion of CO_2_ is substantially slower in water than in air. To overcome these problems, most algae develop CO_2_-concentrating mechanisms (CCMs) that actively take up and enrich CO_2_ and HCO_3_^–^ in the pyrenoid, a chloroplast liquid-like non-membranous compartment rich in Rubisco (Hennacy and Jonikas, 2020). The pyrenoid of the algal model *Chlamydomonas reinhardtii* is penetrated by pyrenoid tubules, which are cylindrical structures of thylakoid membranes (Engel et al., 2015). The pyrenoid tubules may facilitate the rapid diffusion of small molecules, such as adenosine triphosphate (ATP) and sugars, between the chloroplast stroma and pyrenoid (Engel et al., 2015). A starch sheath composed of multiple starch granules forms around the pyrenoid in response to CO_2_ limitation (Kuchitsu et al., 1988), which may prevent CO_2_ diffusion from the pyrenoid.

Earlier reports on *C*. *reinhardtii* suggested that flexible CCM systems operate for adaptation to CO_2_ limitation, i.e., low CO_2_ (LC; approximately 0.03%–0.5%) and very low CO_2_ (VLC; < 0.02%) environments (Wang and Spalding, 2014). Under LC conditions, CO_2_ uptake mechanisms are predominantly activated. It has been suggested that the chloroplast protein limiting CO_2_ inducible protein B (LCIB), which structurally resembles a β-type carbonic anhydrase (Jin et al., 2016), is indispensable for the stimulation of CO_2_ uptake under LC conditions (Yamano et al., 2010; Wang and Spalding, 2014). It may be that LCIB captures CO_2_ leaked from the pyrenoid by unidirectionally hydrating CO_2_ to HCO_3_^-^ under LC conditions (Yamano et al., 2022), though recombinant LCIB did not show carbonic anhydrase activity (Jin et al., 2016). LCIB proteins are dispersed uniformly in the chloroplast under LC conditions, whereas they migrate to the pyrenoid periphery under VLC conditions (Yamano et al., 2022). The starch sheath surrounding the pyrenoid is important in the localization of LCIB (Toyokawa et al., 2020). LCIB interacts with its homolog LCIC (Yamano et al., 2010). Additionally, LCIC accumulation is involved in LCIB migration (Yamano et al., 2022). These results suggest that an LCIB–LCIC complex plays a critical role in CCM regulation depending on the CO_2_ concentration.

Although suppressed under LC conditions, HCO_3_^–^ uptake is activated under VLC conditions. The ABC transporter high-light activated3 (HLA3) and anion channel LCIA, which are localized in the plasma membrane and chloroplast envelope, respectively, act cooperatively for the HCO_3_^–^ uptake (Duanmu et al., 2009; Gao et al., 2015; Yamano et al., 2015).

It has been suggested that CCM-assisted carbon fixation is associated with nutrient availability (Raven et al., 2008). For example, a study using *C*. *reinhardtii*, *Chlamydomonas acidophila*, *Chlamydomonas pitschmannii*, and *Scenedesmus vacuolatus* observed different impacts of P limitation on their CCM, such as reduction of CO_2_ and HCO_3_^–^ uptake (Lachmann et al., 2017). Such impacts might be attributed to energy-demanding processes driven by ATP in the CCMs (Su, 2021). Therefore, P uptake capacity is also crucial for the growth performance of microalgae in water systems. In addition, the P content of microalgal biomass may directly affect its effectiveness as a fertilizer, which will be described further below.

## Phosphorus accumulation associated with membrane lipid remodeling

Nutrient availability substantially affects microalgal growth and lipid metabolism. Owing to their utility for lipid production, interactions between nutrient acquisition and lipid metabolism in microalgae have been extensively studied (Moore et al., 2013; Yaakob et al., 2021). However, for microalgal application in a wastewater purification system followed by fertilizer use, the lipid-metabolism-dependent nutrient uptake capacity of microalgae is of greater interest.

P starvation induces membrane lipid remodeling from phospholipids (e.g., phosphatidylethanolamine, phosphatidylcholine, and phosphatidylglycerol) to non-P-containing glycolipids (e.g., sulfoquinovosyldiacylglycerol, SQDG) and/or betaine lipids (e.g., diacylglyceroltrimethylhomoserine, DGTS), thus facilitating P reallocation to other biochemical and cellular processes (Moseley and Grossman, 2009; Rouached et al., 2010). In *Nannochloropsis oceanica*, the breakdown of phospholipids and the synthesis of DGTS and SQDG are stimulated in the exponential growth phase under P limitation (Mühlroth et al., 2017). Additionally, acyl-editing-mediated conversion of phospholipids to non-P-containing lipids is upregulated in the stationary growth phase (Mühlroth et al., 2017).

The lipid-remodeling-associated P uptake capacity is different in taxonomically diverse microalgae. For example, high P uptake occurs in *Nannochloropsis gaditana*, *Tetraselmis suecica*, and *Picochlorum atomus*, which can actively counterbalance phospholipids with betaine (non-P-containing) lipids under P limitation (Cañavate et al., 2017a; Cañavate et al., 2017b). Meanwhile, such high P uptake is practically absent in *Rhodomonas baltica*, *Chroomonas placoidea*, and *Chaetoceros gracilis*, which constitutively produce betaine lipids with fluctuating abundances of phospholipids depending on P supply levels (Cañavate et al., 2017a; Cañavate et al., 2017b). The diversity may be associated with distinct strategies of microalgae for adaptation to P limitation. Microalgal species displaying a high capacity for P uptake might be useful for the applications in P recycling from wastewater to farmland.

Given that P limitation induces membrane lipid remodeling also in land plants (Nakamura, 2013; Tawaraya et al., 2018; Hayes et al., 2022), information on the molecular mechanisms involved in microalgal lipid remodeling may be beneficial to enhance our understanding of low-P adaptation in land plants. The MYB transcription factor phosphorus starvation response1 (PSR1), a homolog of *Arabidopsis thaliana* phosphate starvation response regulator1 (PHR1), acts as a crucial regulator of the acquisition and reallocation of P in *C. reinhardtii* (Shimogawara et al., 1999; Wykoff et al., 1999; Bajhaiya et al., 2016). In a *N*. *oceanica* mutant deficient in the gene encoding PSR1, low-P-induced replacement of phospholipids with DGTS and SQDG is not observed (Murakami et al., 2020), further supporting the association of PSR1 with low-P-induced membrane lipid remodeling in microalgal species. In addition, the MYB transcription factor lipid remodeling regulator1 (LRL1), a homolog of AtMYB65 from *A*. *thaliana*, upregulates the expression of the gene encoding sulfoquinovosyl diacylglycerol2 (SQD2) involved in SQDG biosynthesis at an advanced stage of the low-P response of *C. reinhardtii* (Hidayati et al., 2019).

## Applications of microalgae for nutrient recycling

Given the aforementioned physiological characteristics that support biomass productivity and nutrient uptake capacity, microalgae are a viable renewable and eco-friendly alternative for conventional wastewater treatment systems (**Table 1**). For example, *Chlorella vulgaris* and *Microcystis* sp. can recover 33 mg P L^-1^ (79%) and 37 mg P L^-1^ (88%), respectively, from an initial concentration of 41 mg P L^−1^ in wastewater in 14 days (Chu et al., 2021). With the escalation in the flow of P from terrestrial to water systems with increased industrialization (Liu et al., 2008; Schlesinger, 2012; Van Dijk et al., 2016), P recovery from wastewaters has become a mandatory practice (Peng et al., 2018). A large amount of P has been recovered annually from wastewater using microalgal biofilm techniques (Sukačová et al., 2020).

**Table 1:** Biomass productivity and nutrient uptake capacity of microalgae in aquatic systems.

Microalgal species	Growth medium	Dry biomass yield (g L^-1^)	Nutrient uptake (mg L^-1^)	Reference
*Chlamydomonas reinhardtii*	BG11	0.4	not described	Farid et al., 2019
*Chlorella minutissima*	wastewater	0.4	N 26.4 P 4.4 K 2.2	Sharma et al., 2021
*Chlorella vulgaris*	BG11	0.8	not described	Dineshkumar et al., 2018
*Chlorella vulgaris*	BG11	0.2	not described	Farid et al., 2019
*Chlorella vulgaris*	wastewater	1.1	N 139.5 P 32.5	Chu et al., 2021
*Chlorella sorokiniana*	BG11	0.2	not described	Farid et al., 2019
*Dunaliell salina*	BG11	0.4	not described	Farid et al., 2019
*Microcystis* sp.	wastewater	1.1	N 161.2 P 36.5	Chu et al., 2021
*Monoraphidium* sp.	diluted anaerobic liquid digestate	0.7-0.8	N-NH_4_^+^ 16-32^※^ P-PO_4_^3–^ 0.8-2.3^※^	Jimenez et al., 2020
*Spirulina platensis*	Zarrouk	1.9	not described	Dineshkumar et al., 2018
				
microalgal consortia (*Scenedesmus* sp. & *Chlorella* sp.)	wastewater	1.8	Protein 175	Silambarasan et al., 2021
microalgal consortia (*Scenedesmus* sp. & *Chlorella* sp.)	wastewater	not described	N-NH_4_^+^ 4.7 P-PO_4_^3–^ 2.3	Avila et al., 2022

^※^ *Monoraphidium* sp. removed 100% (ca. 16-32 mg L^-1^) of N-NH_4_^+^ and 46.6-78.5% (ca. 0.8-2.3 mg L^-1^) of P-PO_4_^3-^ from the diluted anaerobic liquid digestate (Jimenez et al., 2020).

Further utilization of microalgal biomass recovered from wastewater treatment systems may facilitate the establishment of nutrient recycling (**Table 2**). The application of dried microalgal biomass can significantly increase total or plant-available nutrients (Dineshkumar et al., 2018; Dineshkumar et al., 2019; Saadaoui et al., 2019; Sharma et al., 2021) and organic carbon (Renuka et al., 2017) in soils. Deoiled dry biomass, which can be obtained as a residue of microalga-based oil production, improves crop productivity when used as a partial substitute for chemical fertilizers (Silambarasan et al., 2021). There are also reports on the positive effects of microalgal extracts and hydrolysates as a seed primer, foliar spray, and liquid fertilizer (Plaza et al., 2018; Kholssi et al., 2019; Supraja et al., 2020; Kusvuran, 2021). Interestingly, the potential of living microalgae to alleviate saline–alkaline stresses (Ma et al., 2022) and that of a soil-surface biofilm to suppress N loss through NH_3_ volatilization (de Siqueira Castro et al., 2017) have been reported. Circular economy projects using microalgae for wastewater purification and farmland fertilization in a cattle farm (Lorentz et al., 2020) and winery company (Avila et al., 2022) have been tested.

**Table 2:** Effects of microalga-based fertilizers on agricultural crops.

Microalgal species	Application forms	Nutrient content (%)	Crops	Effects	Reference
*Arthrospira platensis, Dunaliella salina*, & *Porphyridium* sp.	extracts (crude polysaccharides)	not described	*S*. *lycopersicum*	plant growth ↑; node number ↑	Rachidi et al., 2020
*Asterarcys quadricellulare*	extracts	not described	*S*. *tuberosum*	potato yield ↑; plant growth ↑; plant chlorophyll, amino acid, & sugar contents ↑; plant nitrate reductase enzyme activity↑; plant nitrogen assimilation ↑	Cordeiro et al., 2022
*Chlorella minutissima*	dried biomass	N 6.0 P 1.0 K 0.5	*Z. mays* & *S. oleracea*	soil nutrient content ↑; plant growth ↑ (vs. chemical fertilizer alone)	Sharma et al., 2021
*Chlorella minutissima*	dried biomass	N 6.0	*S. oleracea*	soil nitrate leaching ↓; leaf N content ↑ (vs. chemical fertilizer alone)	Sharma et al., 2022
*Chlorella sorokiniana*	dried biomass	N 6.1 P 1.2 K 8.9	*H. vulgare*	grain yield ↑ (vs. chemical fertilizer alone)	Suleiman et al., 2020
*Chlorella vulgaris*	hydrochar	N 6.2 P 4.3 K 0.9	*T. aestivum*	soil available P content ↑; plant P use efficiency ↑ (vs. chemical fertilizer alone)	Chu et al., 2021
*Chlorella vulgaris*	extracts	not described	*B. oleracea*	plant growth ↑; plant nutrient content ↑; plant phenolics & flavonoid contents ↑; plant antioxidant activity ↑ (under drought stress)	Kusvuran, 2021
*Chlorella vulgaris*	extracts	N 0.4 K 0.7	*S. lycopersicum*	fruit size ↑; fruit water content ↑; fruit soluble solid content ↑; fruit soluble sugar content ↑; fruit protein content ↑; fruit P, K, Ca, & Mg contents ↑	Suchithra et al., 2022
*Chlorella vulgaris*, *Chlorella sorokiniana*, & *Chlamydomonas reinhardtii*	extracts (crude polysaccharides)	polysaccharides 5.6-8.4	*S. lycopersicum*	plant β-1,3-glucanase activity ↑; plant phenylalanine ammonia lyase activity ↑; plant antioxidant activity ↑; plant fatty acid content ↑	Farid et al., 2019
*Dunaliella salina*	extracts (crude polysaccharides)	polysaccharides 199.8	*S. lycopersicum*	plant lipoxygenase activity ↑	Farid et al., 2019
*Microcystis* sp.	hydrochar	N 8.8 P 5.8 K 0.8	*T. aestivum*	soil available P content ↑; plant P use efficiency ↑; grain yield ↑ (vs. chemical fertilizer alone)	Chu et al., 2021
*Monoraphidium* sp.	dried biomass	N 3.3 P 0.9 K 0.5	*S. lycopersicum*	soil nitrate leaching ↓; plant growth → (vs. chemical fertilizer alone)	Jimenez et al., 2020
*Nannochloropsis oculata*	dried biomass	N 8.1 P 1.3 K 1.4	*S. lycopersicum*	leaf N & P contents →; fruit sugar content →; fruit carotenoid content → (vs. chemical fertilizer alone)	Coppens et al., 2016
					
*Scenedesmus* sp.	extracts	N 8.1 P 2.7 K 0.7	*T. aestivum*	plant nutrient uptake ↑; plant growth → (vs. chemical fertilizer alone)	Shaaban et al., 2010
*Scenedesmus* sp	dried biomass (deoiled)	N 7.5 P 1.6 K 0.7	*O*. *sativa*	plant growth ↑; tillering rate ↑; grain yield ↑ (vs. chemical fertilizer alone)	Nayak et al., 2019
*Spirulina platensis*	extract	N 7.8 P 0.8 K 1.6	*E*. *sativa*, *A. gangeticus*, *B. rapa*, & *B. oleracea*	plant growth →; seedling dry weight → (vs. chemical fertilizer alone)	Wuang et al., 2016
*Tetraselmis* sp.	dried biomass	N 3.4 P 0.5 K 0.5	*P. dactylifera*	soil nutrient content →; plant growth →; plant chlorophyll content →; plant antioxidant activity → (vs. chemical fertilizer alone)	Saadaoui et al., 2019
microalgal bacterial flocs (*Klebsormidium* sp. & *Ulothrix* sp. are dominant)	dried biomass	N 2.4 P 0.6 K 0.2	*S. lycopersicum*	leaf N & P contents →; fruit sugar content →; fruit carotenoid content → (vs. chemical fertilizer alone)	Coppens et al.,2016
microalgal consortia (*Chlorella vulgaris* is dominant)	biomass	not described	*P. glaucum*	soil NH_3_ volatilization ↓	de Siqueira Castro et al., 2017
microalgal consortia (*Chlorella* sp. & *Scenedesmus* sp.)	dried biomass (deoiled)	N 7.8 P 1.7 K 1.1	*S. lycopersicum*	plant growth ↑; plant nutrient content ↑; plant chlorophyll content ↑; fruit yield ↑ (vs. chemical fertilizer alone)	Silambarasan et al., 2021

Effects of microalga-based fertilizer application are described relative to a control or to those following complete and/or partial replacement with chemical fertilizer (vs. chemical fertilizer alone). Arrows indicate higher (↑), lower (↓), and comparable levels (→) of plant or soil parameters.

It has also been reported that algal–bacterial aerobic granular sludge removes greater amounts of P and N from wastewater than does bacteria alone (Wang et al., 2021). Bacterial degradation of organic carbon may mitigate the issue of microalgal CO_2_ acquisition in water systems, which was mentioned above. Additionally, the artificial augmentation of CO_2_ in wastewater via supplementation with flue gas from combustion may also stimulate microalgal biomass productivity and nutrient uptake capacity, potentially resulting in enhanced nutrient recycling (He et al., 2012; Lara-Gil et al., 2016; Yadav et al., 2019).

## Characteristics of microalga-based fertilizers

The application of dry biomass from *Chlorella minutissima* reduced the leaching of nitrate from farmland and increased leaf N content of spinach (*Spinacia oleracea*) plants (Sharma et al., 2022) (**Table 2**). The application of *Asterarcys quadricellulare* extracts significantly stimulated N assimilation and the nitrate reductase activity of potato (*Solanum tuberosum*) plants (Cordeiro et al., 2022). The applications of *C*. *vulgaris* biomass and chemical fertilizer resulted in comparable levels of shoot N uptake in wheat (*Triticum aestivum*) plants (Schreiber et al., 2018). These results demonstrate the effectiveness of the microalga-based fertilizer. However, the level of shoot P uptake was lower in the wheat plants grown under the microalgal treatment than in those grown under the chemical fertilizer treatment (Schreiber et al., 2018), suggesting that microalgal biomass acts as a slow-release P fertilizer. Microalgae can store P as polyphosphates (Delgadillo-Mirquez et al., 2016; Solovchenko et al., 2019; Chu et al., 2021), which are degraded slowly by soil microbes (Powell et al., 2011; Ray et al., 2013; Solovchenko et al., 2019). Furthermore, hydrothermal carbonization of microalgal biomass enhances its characteristics as a slow-release fertilizer, which increases the amount of moderately available P in soils more persistently compared with chemical fertilizer (Chu et al., 2021) (**Table 2**). Such fertilizer characteristics might increase the nutrient use efficiency of crops and/or reduce environmental pollution by suppressing the leaching of nutrients from farmland (Coppens et al., 2016; Jimenez et al., 2020; Sharma et al., 2022).

The application of microalgal extracts enriches essential macronutrients such as P, potassium, calcium, and magnesium in tomato plants (Suchithra et al., 2022) (**Table 2**). Microalga-based fertilizers also supply essential micronutrients as well as beneficial elements for plants (de Haes et al., 2012; Maurya et al., 2016; Wuang et al., 2016; Silva et al., 2019). In a wheat cultivation test, the application of microalgal biomass increased the contents of zinc, iron, copper, and manganese in plants (Rana et al., 2012; Prasanna et al., 2013; Renuka et al., 2017). Microalgal biomass rich in selenium, a beneficial element for plants, has been also suggested to serve as an effective fertilizer (Han et al., 2020).

Garcia-Gonzalez and Sommerfeld (2016) and Deepika and MubarakAli (2020) mentioned the occurrence in microalgal extracts of phytohormones that upregulate plant growth. It has been considered that microalgal components, including phytohormones, stimulate the production of antifungal substances in plants (Spolaore et al., 2006; Coppens et al., 2016). In addition, crude polysaccharides obtained from microalgae have a biostimulant-like effect on plants (Farid et al., 2019; Rachidi et al., 2020) (**Table 2**). Plant morphological traits, such as plant height, leaf number, tillering rate, root length, and lateral root number, are positively affected by the application of a microalga-based fertilizer depending on its dosage (Wuang et al., 2016; Nayak et al., 2019; Deepika and MubarakAli, 2020) (**Table 2**). Commercially important components of fruit, such as carotenoids and sugars, increase in response to the application of microalga-based fertilizers (Kumari et al., 2011; Coppens et al., 2016; Mutale-Joan et al., 2020; Cordeiro et al., 2022). These changes might be partially due to the effect of plant growth regulators in microalgal biomass, although further investigation is required for verification.

## Conclusions and prospects

To achieve rapid growth and efficient nutrient accumulation in water systems, microalgae developed mechanisms such as flexible CCMs and membrane lipid remodeling. Previous research has shed light on the sophisticated molecular interactions underlying the physiological characteristics of microalgae, which support its utility as a wastewater purification system and fertilizer. Applications of microalgae in a wastewater purification system followed by fertilizer use may facilitate the establishment of nutrient recycling. Many studies have shown that application of microalgal biomass can provide nutrients essential for plants and enrich organic carbons in soils. In addition, microalgal biomass contains slowly degradable forms of plant-essential nutrients, reducing the leaching of the nutrients from farmland. Furthermore, microalga-based fertilizers are regarded as suppliers of plant growth regulators. However, challenges remain in the expansion of microalga-based technologies. For example, a life cycle assessment highlighted the detrimental impact of electricity consumption required for microalgal cultivation (Diniz et al., 2017; de Souza et al., 2019). In addition, the application of a microalga-based fertilizer can stimulate the emission of greenhouse gases, such as N_2_O and CO_2_, from soils (Suleiman et al., 2020). Thus, further technological advances, as well as a more in-depth understanding of microalgal physiology, are required for wider implementation of microalgal applications for sustainable agriculture.

## Author contributions

IC, TM, and NO wrote the manuscript. All authors contributed to the article and approved the submitted version.

## Funding

This work was partially supported by the Japan Society for the Promotion of Science (JSPS) KAKENHI grant (JP#22K14876).

## Conflict of interest

The authors declare that the research was conducted in the absence of any commercial or financial relationships that could be construed as a potential conflict of interest.

## Publisher’s note

All claims expressed in this article are solely those of the authors and do not necessarily represent those of their affiliated organizations, or those of the publisher, the editors and the reviewers. Any product that may be evaluated in this article, or claim that may be made by its manufacturer, is not guaranteed or endorsed by the publisher.
